# Intraventricular hemorrhage in preterm newborns: a multicenter study in four Brazilian hospitals

**DOI:** 10.1016/j.jped.2026.101574

**Published:** 2026-06-15

**Authors:** Gabriel Fernando Todeschi Variane, Danieli Mayumi Kimura Leandro, Silvia Schoenau de Azevedo, Marcelo Jenné Mimica, Maurício Magalhães, Krisa Page Van Meurs, Valerie Y. Chock

**Affiliations:** a Irmandade da Santa Casa de Misericórdia de São Paulo, Department of Pediatrics, Division of Neonatology, São Paulo, SP, Brazil; b Protecting Brains & Saving Futures, São Paulo, SP, Brazil; c Santa Casa de São Paulo, Faculdade de Ciências Médicas, São Paulo, SP, Brazil; d Stanford University School of Medicine, Division of Neonatal and Developmental Medicine, Stanford, United States; e Lucile Packard Children’s Hospital Stanford, Palo Alto, CA, United States

**Keywords:** Cerebral intraventricular hemorrhage, Infant, premature, Cranial ultrasonography, Body temperature regulation

## Abstract

**Objective:**

To identify the incidence, demographic characteristics, and risk factors for intraventricular hemorrhage (IVH) in preterm infants admitted to Brazilian neonatal intensive care units (NICUs).

**Methods:**

This prospective, observational cohort study was conducted over a one-year period in four NICUs in Brazil. All newborns with gestational age (GA) < 32 weeks or birth weight (BW) < 1500 g, born between September 2023 and September 2024, were included. Demographic data and short-term outcomes were collected. Multinomial logistic regression was performed to evaluate associations between clinical variables and IVH severity.

**Results:**

A total of 268 newborns were enrolled. The mean BW and GA were 1138 g (SD ±388 g) and 29 weeks and 1 day (SD ±3 weeks), respectively. Normal cUS were seen in 54.1%, mild IVH in 20.5%, severe IVH in 10.4%, and 8.2% died prior to a cUS being performed. Infants with the outcome of severe IVH or death prior to cUS exhibited lower BW, GA, Apgar scores, rates of cesarean section, fewer complete courses of antenatal steroids, and were more likely to have undergone advanced resuscitation in the delivery room and significant interventions in the early neonatal period. Hypothermia was prevalent across all groups. Infants with severe IVH had significantly higher rates of death prior to hospital discharge and longer length of stay.

**Conclusions:**

Identifying risk factors for IVH is essential for developing strategies to optimize outcomes. Implementation of a standardized IVH prevention bundle in Brazilian NICUs focusing on factors shown to adversely affect outcomes is warranted.

## Introduction

Intraventricular hemorrhage (IVH) is a common and severe complication of prematurity due to the fragility of the germinal matrix and the immature cerebrovascular system^1^ and remains a significant cause of morbidity and mortality, particularly in infants born before 32 weeks of gestation or weighing <1500 *g*.^2^ Despite substantial advances in neonatal care, IVH continues to affect up to 50% of very low birth weight infants in many settings, with severe grades of IVH strongly associated with poor neurodevelopmental outcomes, such as cerebral palsy, cognitive impairment, and sensory deficits.^3^^,^^4^ IVH is a multifactorial condition influenced by antenatal, perinatal, and postnatal factors, including antenatal exposure to steroids, mode of delivery, tracheal intubation, and inotropic use.^5^^,^^6^

Most epidemiological data on IVH originate from high-income countries with well-established neonatal networks with consistent quality of care and distinct population characteristics. However, in low- and middle-income countries (LMICs), the incidence and outcomes of IVH may differ due to resource constraints, limited access to specialized neonatal care, and disparities in the implementation of evidence-based practices.^7^

Brazil, as an LMIC with heterogeneity in perinatal care, offers a unique setting to understand the burden of IVH in preterm neonates. The Brazilian Neonatal Research Network has documented that 30.4% of preterm infants in Brazil experience IVH, with significant variations in severity and outcomes across neonatal intensive care units (NICUs). Moreover, trends from 2013 to 2018 indicate an increasing incidence of IVH, reflecting the increased rate of survival of lower GA infants, the complexity of neonatal care, and possible gaps in resource allocation and clinical protocols.^8^

To address this knowledge gap, the authors conducted a multicenter cohort study in four Brazilian NICUs, including both private and public centers, to identify the rates and severity of IVH among infants born at < 32 weeks’ gestation or < 1500 g, and secondarily, to identify the demographic, maternal, prenatal, delivery, and neonatal risk factors for IVH in these Brazilian NICUs.

## Methods

This observational, prospective cohort study was conducted in four NICUs in Brazil from September 2023 to September 2024. The study was approved by the Research Ethics Committees of all participating hospitals and followed the STROBE (Strengthening the Reporting of Observational Studies in Epidemiology) guideline.

One center was based in the state of Goiás (Central-West region) and three in São Paulo (Southeast region). Brazil’s healthcare system is characterized by a dual structure with both public and private sectors. Of the four participating NICUs, three were public hospitals integrated into the Brazilian Unified Health System (Sistema Único de Saúde – SUS), and one was a private facility. All centers are referral institutions for high-risk pregnancies and provide level III neonatal care. The inclusion of centers from different geographic regions and with distinct organizational structures and resource availability was intentional, aiming to capture the heterogeneity of neonatal care in Brazil and enhance the external validity of the findings. Prior to the study initiation, training sessions were held with the center’s investigators to standardize the data collection process. A shared protocol and centralized database were used to ensure data consistency across centers.

The study population consisted of preterm infants born at < 32 weeks’ gestation or with birth weight < 1500 g, admitted to the participating NICUs during the one-year study period. Infants with congenital malformations or genetic syndromes were excluded.

Antenatal, in-hospital, and outcome data were collected from the patients' medical records, including information on invasive interventions in the NICU, such as the use of sedatives or analgesics, inotropes, fluid bolus, and mechanical ventilation. Antenatal steroid exposure was categorized as complete when two doses were administered, irrespective of the time interval between doses. Exposure was considered incomplete when fewer than two doses were administered, regardless of timing. The most severe IVH grade identified on cranial ultrasound (cUS) during NICU stay was included in the analysis. The diagnosis and classification of IVH were based on Papile’s classification system.^9^ Grades I and II IVH were considered to be mild IVH, and grades III and IV were considered to be severe IVH.^9^ Post-hemorrhagic ventricular dilatation was also classified as a severe finding.

Descriptive analyses were conducted using frequencies for categorical variables and means, medians, standard deviations, and interquartile ranges for continuous variables, according to data distribution. The results of cUS were divided into normal (no evidence of IVH), mild, and severe. To avoid exclusion of high-risk infants, those who died before cUS assessment were included in a composite outcome of severe IVH or death prior to cUS. Infants with leukomalacia and no IVH were excluded from this comparison.

Multinomial logistic regression was performed to evaluate associations between clinical variables and IVH severity. Crude associations were estimated using univariate models. Subsequently, domain-specific multivariable models were constructed a priori based on clinical plausibility. A baseline demographic model included gestational age (GA), sex, mode of delivery, and hospital sector. Separate models evaluated prenatal exposures, perinatal variables, and early postnatal interventions within the first 72 h of life, all adjusted for baseline variables. GA and birth weight were not included simultaneously due to their biological collinearity. Multicollinearity was assessed using the variance inflation factor (VIF), and variables with VIF > 5 were excluded. Results are presented as relative risk ratios (RRR) with 95% confidence intervals (CI), and statistical significance was defined as p < 0.05.

## Results

### *Baseline characteristics*

A total of 268 preterm infants were included in the study, and 142 (53%) were male. The mean birth weight was 1138 g (SD ±388 g), and the mean GA was 29 weeks (SD ±3). Cesarean sections accounted for 163 (61%) deliveries, and 247 (92%) neonates were inborn. A detailed flowchart on patient selection is presented in Supplemental Figure 1, and baseline characteristics are described in Table 1.

**Table 1:** Baseline characteristics.

Variables	All Subjects N = 268	N total (cUS + death) = 250		
		Normal cUS N = 145 (58%)	Mild IVH N = 55 (22%)	Severe IVH OR death N = 50 (20%)
Public	190 (70.9)	97 (66.9)	38 (69.1)	42 (84.0)
Private	78 (29.1)	48 (33.1)	17 (30.9)	8 (16.0)
Male, n (%)	142 (53.0)	74 (51.0)	30 (54.5)	28 (56.0)
Cesarean section, n (%)	163 (61.0)	100 (69.0)	29 (52.7)	24 (48.0)
Birth weight, grams, mean (SD)	1138 (±388)	1220 (±342)	1150 (±391)	884 (±383)
Gestational age, weeks, mean (SD)	29 1/7 (±3 1/7)	29 6/7 (±2 6/7)	29 1/7 (±2 6/7)	26 4/7 (±3 0/7)
Apgar 1′, median (IQR)	6 (4–8)	7 (5–8)	6 (5–8)	3 (2–6)
Apgar 5′, median (IQR)	8 (7–9)	9 (8–9)	8 (8–9)	7 (5–8)
Apgar 10′, median (IQR)	8 (7–9)	9 (8–9)	8 (7–9)	7 (6–8)

### *Antenatal care and delivery room events*

Only 126 newborns (47%) were exposed to a complete course of antenatal steroids, 69 (25.7%) to a partial course, and 134 (50%) were exposed to antenatal magnesium sulfate. During the delivery room resuscitation, 57 (21.3%) received only positive pressure ventilation, 107 (39.9%) received endotracheal intubation, and 8 (3.0%) received chest compressions. Hypothermia, defined as a temperature < 36.5° C, occurred in 131 (49.0%) of the neonates in the delivery room. Detailed information on antenatal care and delivery room events is shown in Table 2.

**Table 2:** Antenatal and delivery room events in the study population.

Variables	All Subjects No = 268	N total (cUS + death) = 250		
		cUS Normal No = 145 (58%)	Mild IVH No = 55 (22%)	Severe IVH OR death No = 50 (20%)
**Antenatal care**				
Antenatal steroids:				
Complete course, n (%)	126 (47.0)	77 (53.1)	24 (43.6)	16 (32.0)
Partial steroids, n (%)	69 (25.7)	35 (24.1)	14 (25.5)	16 (32.0)
No steroids, n (%)	73 (27.2)	33 (22.8)	17 (30.9)	18 (36.0)
Antenatal magnesium sulfate, n (%)	134 (50)	72 (49.7)	29 (52.7)	25 (50.0)
**Delivery room events**				
Hypothermia (T < 36 °C) in the delivery room, n (%)	131 (49.0)	72/115 (62.6)	23/40 (57.5)	25/33 (75.8)
Positive pressure ventilation only, n (%)	57 (21.3)	38 (26.2)	10 (18.2)	5 (10)
Positive pressure ventilation + Intubation, n (%)	107 (39.9)	47 (32.4)	17 (30.9)	34 (68.0)
Positive pressure ventilation + intubation + Chest compressions, n (%)	8 (3.0)	2 (1.4)	2 (3.6)	4 (8.0)
**Neonatal care in the first 72 h after birth**				
Caffeine, n (%)	224 (84.0)	124 (85.5)	50 (90.9)	35 (70.0)
Sedation or analgesic, n (%)	55 (20.5)	22 (15.2)	14 (25.4)	19 (38)
Mechanical ventilation, n (%)	143 (53.3)	60 (41.4)	28 (50.9)	45 (90.0)
Parenteral nutrition, n (%)	233 (87.0)	129 (89.0)	52 (94.5)	38 (76.0)
Fluid bolus, n (%)	56 (21.0)	21 (14.5)	14 (25.5)	19 (38.0)
Inotropes, n (%)	68 (25.0)	18 (12.4)	15 (27.3)	32 (64.0)
Parental touch, n (%)	223 (84.0)	125 (86.2)	47 (85.5)	36 (72.0)
Minimal handling, n (%)	207 (77.0)	113 (77.9)	39 (70.9)	41 (82.0)
Hypothermia at NICU admission, n (%) n = 144	177 (66.0)	87/143 (60.8)	38/55 (69)	40/50 (80)
Hypothermia within 72 h after admission, n (%)	236 (88.0)	126/143 (88.1)	49/55 (89.1)	46/48 (95.8)

### *First 72 h of life*

Within the first 72 h, 84% (n = 224) of the infants were exposed to caffeine, 20.5% (n = 55) sedatives or analgesics, 87% (n = 233) parenteral nutrition, 21% (n = 56) fluid bolus, and 25% (n = 68) inotropes. Mechanical ventilation was used in 53.3% (n = 143). Parental touch was applied to 84.0% (n = 223) of newborns, and 77.0% (n = 207) were cared for using minimal handling protocols. Hypothermia was observed in 66.0% (n = 177) of newborns at NICU admission and in 88.0% (n = 236) within the first 72 h.

### *Cranial ultrasound findings and in-hospital outcomes*

**Figure 1:**
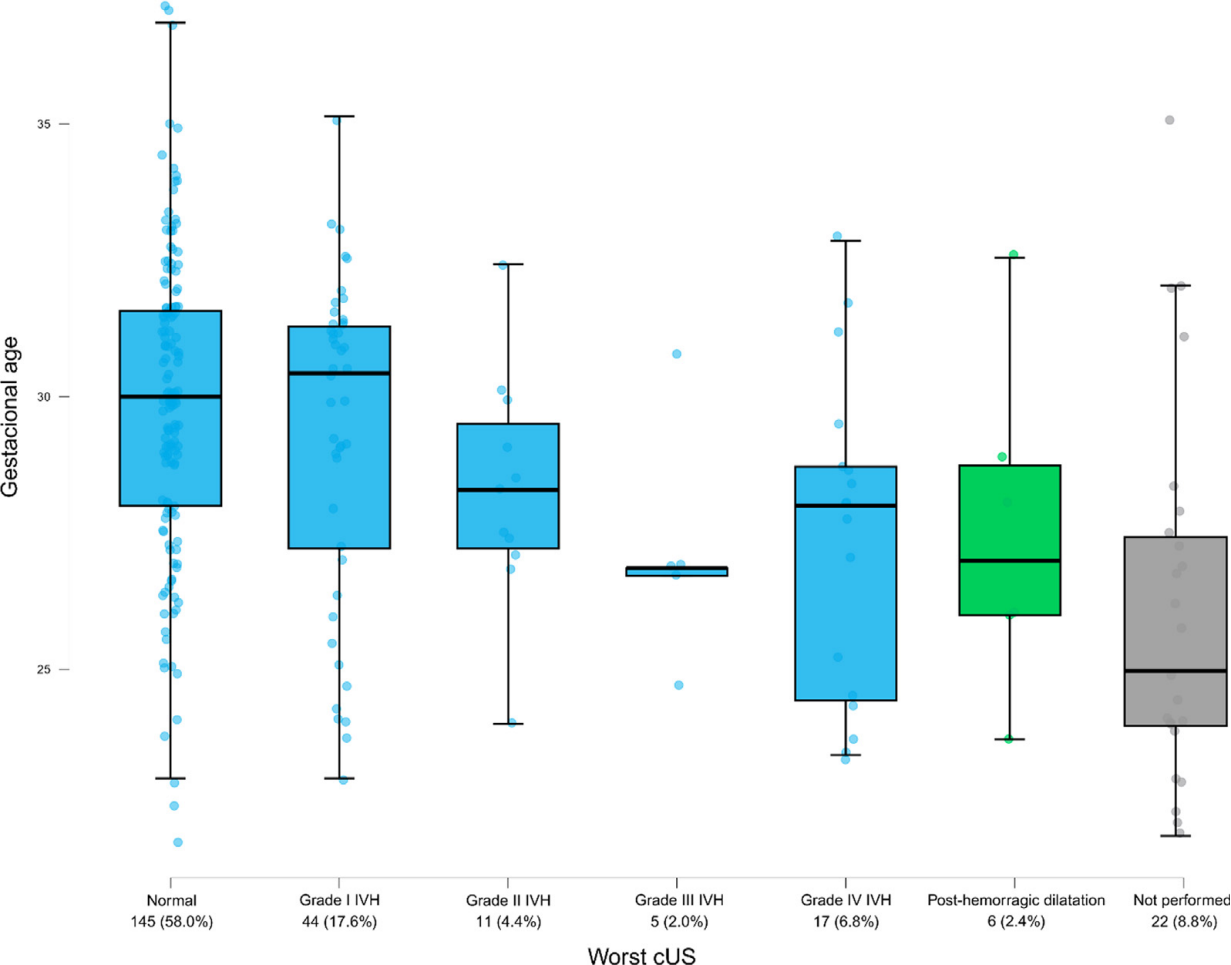
cUS findings during hospitalization by gestational age. The box shows where most values are concentrated, while the whiskers show the typical range of the data. Points beyond the whiskers represent more extreme values.

During NICU stay, 246 (91.7%) infants underwent cUS, and 22 (8.2%) patients died prior to performing a cUS. The median days to death for these patients was 2 days (IQR 0–4). Among the patients that underwent cUS, the first exam was performed at a median of 5 days of life (DOL) (IQR 3–6), and the worst exam was documented at a median of 14 DOL (IQR 5–29). Characteristics and incidence of cUS findings are shown in Figure 1.

In survivors, a normal cUS was observed in 145 (59%) infants, mild IVH in 55 (22.4%), severe IVH in 28 (11.4%), and leukomalacia with no IVH in 18 (7.3%). Infants with severe IVH or death tended to have lower birth weight and GA and lower Apgar scores. Cesarean delivery and exposure to a complete course of antenatal steroids were less frequent in infants with severe IVH or death, whereas intubation in the delivery room was more common. Antenatal and delivery room data are displayed in Table 2.

In the first 72 h after birth, infants with the combined outcome of death or severe IVH were less frequently exposed to caffeine and more frequently exposed to sedative or analgesics, mechanical ventilation, inotropes, and fluid boluses. Detailed information on the neonatal care in the first 72 h after birth is summarized in Table 2.

Hypothermia was common in the delivery room (49.0%), at NICU admission (66.0%), and in the first 72 h after admission (88.0%). Although hypothermia appeared more frequently among infants with severe IVH, differences between groups were modest (Table 2).

Infants with severe IVH had higher rates of death prior to hospital discharge, with a median age at death of 32 days (IQR 18–40). Length of stay among survivors was also longer in infants with severe IVH, with a median of 95 days (IQR 51–114).

In univariate analyses (Table 3), lower GA and birth weight, advanced resuscitation in the delivery room, mechanical ventilation, endotracheal intubation, sedation or opioid use, inotrope use, and fluid bolus administration were associated with increased relative risk of severe IVH or death. Cesarean delivery was associated with a lower relative risk of any IVH or death.

**Table 3:** Univariate logistic regression analysis.

Variables	IVH severity	RRR (95% CI)	p-value
Private Sector	Mild	0.90 (0.46 – 1.76)	0.767
	Severe / Death	0.44 (0.16 – 1.23)	0.117
Cesarean delivery	Mild	0.50 (0.27 – 0.95)	0.034
	Severe / Death	0.34 (0.15 – 0.77)	0.010
Gestational age	Mild	0.91 (0.82 – 1.01)	0.084
	Severe or death	0.68 (0.60 – 0.77)	<0.001
Birth weight	Mild	1.00 (0.99 – 1.00)	0.231
	Severe or death	1.00 (0.996 – 0.998)	<0.001
Female sex	Mild	0.87 (0.47 – 1.62)	0.657
	Severe / Death	0.67 (0.30 – 1.54)	0.350
Complete course of antenatal corticosteroid	Mild	0.61 (0.29 – 1.27)	0.185
	Severe / Death	0.59 (0.22 – 1.60)	0.299
Magnesium sulfate	Mild	1.13 (0.61 – 2.11)	0.698
	Severe / Death	0.88 (0.39 – 1.98)	0.755
Advanced resuscitation in DR	Mild	0.86 (0.46 – 1.61)	0.641
	Severe / Death	18.00 (2.38 – 136.15)	0.005
Mechanical Ventilation	Mild	1.47 (0.79 – 2.74)	0.227
	Severe / Death	18.42 (4.21 – 80.56)	<0.001
Orotracheal intubation	Mild	1.55 (0.83 – 2.89)	0.172
	Severe / Death	32.32 (4.28 – 244.24)	0.001
Caffeine	Mild	1.69 (0.61 – 4.74)	0.316
	Severe / Death	1.41 (0.39 – 5.10)	0.599
Sedation or Opioids	Mild	1.91 (0.89 – 4.07)	0.094
	Severe / Death	3.11 (1.27 – 7.61)	0.013
Use of inotropes	Mild	2.65 (1.22 – 5.73)	0.013
	Severe / Death	9.41 (3.84 – 23.06)	<0.001
Volume expansion	Mild	2.02 (0.94 – 4.32)	0.072
	Severe / Death	3.82 (1.57 – 9.29)	0.003
Positive touch	Mild	0.94 (0.39 – 2.28)	0.891
	Severe / Death	0.96 (0.30 – 3.06)	0.945
Minimal handling	Mild	0.69 (0.34 – 1.39)	0.301
	Severe / Death	1.70 (0.55 – 5.25)	0.357
Hypothermia in the DR	Mild	0.81 (0.39 – 1.68)	0.568
	Severe / Death	1.49 (0.54 – 4.14)	0.441
Hypothermia within 72 h after admission	Mild	1.10 (0.41–2.96)	0.847
	Severe / Death	1.75 (0.38–8.06)	0.470

Abbreviations: RRR, Relative risk ratio; CI, Confidence interval; DR, Delivery room.

In multinomial regression models (Table 4), lower GA remained independently associated with severe IVH or death in the baseline and antenatal care models (models 1 and 2). Advanced resuscitation in the delivery room was strongly associated with severe IVH or death in Model 3 (RRR 13.4, 95% CI 2.62–68.50, p = 0.002). In the model evaluating neonatal care (model 4) within the first 72 h of life, the use of inotropes was independently associated with severe IVH or death (RRR 4.14, 95% CI 1.28–13.39, p = 0.018).

**Table 4:** Adjusted multinomial logistic regression models.

Variables	Outcome Category	RRR (95% CI)	p-value
Model 1: Baseline characteristics.			
Private Sector	Mild	1.07 (0.52–2.22)	0.847
	Severe / Death	0.40 (0.13–1.26)	0.116
Cesarean delivery	Mild	0.56 (0.27–1.13)	0.106
	Severe / Death	0.77 (0.29–2.01)	0.590
Gestational age	Mild	0.93 (0.83–1.05)	0.226
	Severe / Death	0.73 (0.61–0.86)	< 0.001
Female sex	Mild	0.89 (0.47–1.67)	0.707
	Severe / Death	0.54 (0.22–1.34)	0.185
Model 2: Antenatal care.			
Private Sector	Mild	0.62 (0.26–1.47)	0.277
	Severe / Death	0.24 (0.05–1.01)	0.051
Cesarean delivery	Mild	0.71 (0.30–1.68)	0.434
	Severe / Death	1.00 (0.29–3.47)	0.997
Gestational age	Mild	0.98 (0.86–1.13)	0.816
	Severe / Death	0.76 (0.62–0.94)	0.012
Female sex	Mild	0.71 (0.34–1.49)	0.371
	Severe / Death	0.64 (0.23–1.84)	0.412
Complete course of antenatal steroids	Mild	0.63 (0.26–1.53)	0.306
	Severe / Death	0.56 (0.15–2.12)	0.392
Magnesium sulfate	Mild	1.30 (0.54–3.14)	0.564
	Severe / Death	1.68 (0.46–6.19)	0.435
Model 3: Delivery room events.			
Private Sector	Mild	0.98 (0.41–2.31)	0.959
	Severe / Death	0.44 (0.11–1.71)	0.236
Cesarean delivery	Mild	0.67 (0.28–1.57)	0.353
	Severe / Death	0.48 (0.15–1.54)	0.218
Gestational age	Mild	0.87 (0.75–1.03)	0.101
	Severe / Death	0.90 (0.73–1.10)	0.285
Female sex	Mild	1.08 (0.51–2.26)	0.845
	Severe / Death	0.70 (0.24–2.10)	0.530
Advanced resuscitation in the DR	Mild	0.96 (0.39–2.39)	0.936
	Severe / Death	13.4 (2.62–68.50)	0.002
Hypothermia in the DR	Mild	0.71 (0.31–1.66)	0.433
	Severe / Death	0.54 (0.16–1.89)	0.338
Model 4: Neonatal care in the first 72 h after birth.			
Private Sector	Mild	1.47 (0.61–3.55)	0.392
	Severe / Death	0.53 (0.11–2.55)	0.430
Cesarean delivery	Mild	0.51 (0.24–1.06)	0.073
	Severe / Death	0.79 (0.27–2.26)	0.654
Gestational age	Mild	0.97 (0.83–1.14)	0.720
	Severe / Death	0.88 (0.71–1.09)	0.248
Female sex	Mild	0.84 (0.42–1.68)	0.620
	Severe / Death	0.59 (0.21–1.63)	0.305
Mechanical ventilation	Mild	0.57 (0.15–2.19)	0.413
	Severe / Death	1.53 (0.20–11.89)	0.686
Orotracheal intubation	Mild	1.41 (0.38–5.23)	0.608
	Severe / Death	9.20 (0.64–131.43)	0.102
Caffeine	Mild	1.54 (0.46–5.11)	0.482
	Severe / Death	0.47 (0.86–2.57)	0.383
Sedation or opioids	Mild	1.64 (0.66–4.08)	0.286
	Severe / Death	1.06 (0.33–3.36)	0.925
Use of inotropes	Mild	2.18 (0.76–6.25)	0.148
	Severe / Death	4.14 (1.28–13.39)	0.018
Fluid bolus	Mild	1.18 (0.45–3.06)	0.737
	Severe / Death	1.08 (0.37–3.17)	0.886
Early nutrition <72h	Mild	1.64 (0.41–6.53)	0.479
	Severe / Death	1.16 (0.09–13.70)	0.905
Positive touch	Mild	1.44 (0.47–4.39)	0.519
	Severe / Death	1.07 (0.20–5.80)	0.940
Minimal handling	Mild	0.49 (0.21–1.15)	0.103
	Severe / Death	1.06 (0.27–4.12)	0.933
Hypothermia within 72h hours after admission	Mild	0.97 (0.34–2.77)	0.951
	Severe / Death	1.06 (0.18–6.26)	0.950

RRR, Relative risk ratio; CI, Confidence interval; DR, delivery room.

Footnote: Multicollinearity was assessed using variance inflation factors (VIF). All variables included in the models presented VIF values < 5.

Reference category = absence of exposure unless otherwise specified.

## Discussion

This prospective multicenter study conducted in four Brazilian NICUs revealed a 36.4% incidence of IVH among preterm infants born at < 32 weeks' gestation or weighing < 1500 g, with the combined outcome of severe IVH or death prior to cUS occurring in 20%. Infants with this combined outcome were characterized by lower birth weight and GA, lower Apgar scores, and lower rates of cesarean delivery, and were less likely to be exposed to a complete course of antenatal corticosteroids and more likely to have undergone advanced resuscitation in the delivery room and more invasive interventions in the NICU, such as use of sedatives or analgesics, inotropes, fluid bolus, and mechanical ventilation. Hypothermia was prevalent across all groups, but particularly elevated among those with severe IVH or death. In multinomial regression analyses, lower GA, advanced resuscitation in the delivery room, and early use of inotropes were independently associated with severe IVH or death.

The IVH rates seen in this study align with global and local reports. A previous meta-analysis of 64 studies including data from 9633 preterm infants ≤ 32 weeks GA born after 2007, reported a global IVH occurrence of 31% (95% CI 25- 36%) and 11% (95% CI 8–14%) of severe IVH.^2^ A multicenter cohort study including very low birth weight newborns < 1500 g, published by the Brazilian Network on Neonatal Research, reported a similar overall rate of IVH in 30.4%, with 9.8% classified as severe.^8^ These findings highlight that a substantial proportion of preterm infants remain at high risk for severe brain injury.

In this analysis, infants with severe IVH or death presented more frequently with lower birth weight, GA, and Apgar scores. In the adjusted multinomial analyses, GA emerged as the most consistent independent predictor of severe IVH or death. These findings are consistent with previously identified risk profiles for adverse neonatal outcomes.^10^^,^^11^ The fragility of the vessels in the germinal matrix at early GA and the instability of cerebral blood flow place preterm newborns at a higher risk for brain injury.^10^

In the studied cohort, cesarean delivery appeared less frequent among infants with severe IVH or death; however, this association was not maintained after adjustment for other perinatal factors. Previous studies have reported conflicting findings regarding the protective role of cesarean delivery in extremely preterm infants.12., 13., 14. While some suggest that cesarean delivery may reduce hemodynamic instability during birth, as vaginal delivery may expose fragile cerebral vessels to significant fluctuations in cerebral blood flow and oxygen saturation, others have shown that the association disappears after adjustment for GA and perinatal condition.13., 14., 15., 16. These findings suggest that the relationship between delivery mode and IVH risk is likely confounded by the clinical circumstances leading to preterm birth rather than reflecting a direct causal effect of delivery method.

While some risk factors for IVH are inherent to the unpredictability of preterm birth, many are modifiable through clinical practice. Of particular concern in the studied cohort were the low rate of antenatal corticosteroid administration (47%), high rates of inotrope use (25%), delivery room intubation (39.9%), and postnatal hypothermia (up to 88% within 72 h), all of which are strongly associated with increased risk for IVH and adverse neonatal outcomes.12., 13., 14., 15., 16., 17.

Previous studies have demonstrated that the use of antenatal corticosteroids significantly reduces the incidence of IVH and improves survival in preterm infants by enhancing pulmonary and cerebrovascular stability.15., 16., 17., 18. As shown in a large population-based cohort from the California Perinatal Quality Care Collaborative (CPQCC), including 44,028 preterm infants, antenatal corticosteroid exposure was the only factor independently associated with a reduced risk of severe IVH across all GA groups.^15^ Although infants exposed to a complete course of antenatal corticosteroids in the present cohort showed lower crude rates of severe IVH or death, this association was not maintained in adjusted analyses. This may reflect the relatively small number of severe IVH cases or residual confounding related to the timing of steroid administration and perinatal condition. Nonetheless, the relatively low rate of complete antenatal corticosteroid exposure observed in this cohort (47%) highlights an important opportunity for improvement in perinatal care.

The need for advanced resuscitation in the delivery room was strongly associated with severe IVH or death in the adjusted analysis. This association likely reflects the severity of cardiorespiratory compromise immediately after birth, which may lead to abrupt fluctuations in cerebral blood flow during the transitional period. Previous observational studies have similarly demonstrated that extensive resuscitation and multiple intubation attempts in the delivery room are associated with increased risk of severe IVH in very preterm infants.^15^^,^^19^ These findings underscore the importance of gentle stabilization strategies and minimizing hemodynamic instability during the immediate postnatal transition.

Similarly, early hemodynamic instability also appears to play a central role in IVH pathogenesis.^17^ In the adjusted models, the use of inotropes within the first 72 h of life remained independently associated with severe IVH or death. Fluctuations in cerebral blood flow and systemic blood pressure during the early neonatal period have been strongly implicated in the development of germinal matrix hemorrhage.19., 20., 21. A previous prospective study of 497 extremely preterm infants (≤ 29 weeks) found that early inotrope use was associated with significantly increased odds of severe brain injury, including IVH of any grade.^21^

Hypothermia was highly prevalent in the studied cohort, occurring in nearly half of the infants in the delivery room and in the majority during the first 72 h after admission. Maintaining normothermia in the immediate postnatal period is critical, as hypothermia is linked to impaired cardiorespiratory transition and increased morbidity in preterm neonates.^22^^,^^23^ A systematic review including over 300,000 very preterm infants reported that admission hypothermia was associated with increased mortality and higher risk of IVH, bronchopulmonary dysplasia, retinopathy of prematurity, and sepsis.^23^ Although hypothermia was not independently associated with IVH severity in these models, its high prevalence underscores the importance of improving thermal management practices in the delivery room and early NICU care.

Taken together, the present findings highlight urgent opportunities for improvement in perinatal and neonatal practices in Brazilian NICUs. Multiple studies have consistently demonstrated that evidence-based interventions significantly reduce the incidence of IVH in preterm infants.^24^^,^^25^ A recognized strategy to mitigate these multifactorial risks is to implement care bundles.^17^^,^26., 27., 28. Rather than focusing on single interventions, bundles combine multiple evidence-based practices and training across the perinatal continuum to maximize neuroprotection. A previously published quality improvement study in Canada has shown a substantial reduction in the use of inotropes, fluid boluses, and opioids. These changes were associated with a 69% decrease in IVH rates.^17^ Increasing the uptake of these evidence-based practices has the potential to yield substantial improvements in survival without severe morbidity among the most vulnerable preterm newborns.^4^^,^^10^^,^^14^

A major strength of this study lies in its prospective design and inclusion of both public and private hospitals in an LMIC. This approach allows for a more comprehensive and realistic representation of neonatal care across different healthcare settings in Brazil, capturing systemic challenges and institutional variability. The multicenter nature and standardized methodology enhance the external validity of the findings and support their application in guiding local and national strategies for IVH prevention in similar contexts. However, the study sample may not fully represent all regions of the country, as differences in NICU resources and clinical practices could affect generalizability.

Nonetheless, certain limitations must be acknowledged. First, as an observational study, causal inferences cannot be firmly established. Second, a significant proportion of infants (8.2%) did not undergo cUS, possibly due to early mortality or limited access to imaging in the first DOL, potentially leading to underestimation of IVH incidence. Third, although several variables traditionally considered risk factors for severe IVH did not reach statistical significance in multinomial analyses, the sample size and number of outcome events may have limited the statistical power to detect modest associations; therefore, these findings should be interpreted with caution. These limitations underscore the need for systemic improvements in education, clinical practice, and access to early neuroimaging in Brazilian NICUs to improve morbidity and mortality due to IVH.

In conclusion, this multicenter cohort study highlights the persistent burden of IVH among preterm infants in Brazilian NICUs and identifies key factors associated with severe IVH or death, including lower GA, advanced delivery room resuscitation, and early hemodynamic instability. These findings support the development and implementation of standardized IVH prevention bundles, focused on optimizing antenatal corticosteroid administration, thermoregulation, gentle resuscitation, and judicious hemodynamic management for preterm infants in Brazil. Future studies should prospectively assess the impact of such interventions on IVH incidence and neonatal outcomes in Brazilian NICUs.

## Funding

This work was supported by the Stanford Center for Innovation in Global Health.

## Data availability

The data that support the findings of this study are available from the corresponding author.

## Conflicts of interest

The authors declare no conflicts of interest.

## Appendix Supplementary materials

Image, application 1
