# Supplementary material for: Intraventricular hemorrhage in preterm newborns: a multicenter study in four Brazilian hospitals
Source: J Pediatr (Rio J). 2026 Jun 15;102(5):101574. doi: 10.1016/j.jped.2026.101574 (PMC13284472; doi:10.1016/j.jped.2026.101574)
Supplement: Supplementary file 1 [file mmc1.docx]

**JPED-D-25-00553_Supplementary Material**

**Supplemental Figure 1** Patient inclusion flowchart.

Total admissions of infants <32 weeks’ gestation or birth weight <1500g

between September 2023 and September 2024

**(N = 268)**

Leukomalacia without IVH **(N = 18)**

Severe IVH **(N = 28)**

Mild IVH

**(N = 55)**

Normal cUS (no evidence of IVH)

**(N = 145)**

Infants who underwent cUS

**(N =246)**

Died before cUS

**(n = 22)**
